# Effect of acceptance and commitment therapy on mood, sleep quality and quality of life in menopausal women: a randomized controlled trial

**DOI:** 10.1186/s12888-022-03768-8

**Published:** 2022-02-11

**Authors:** Zahra Monfaredi, Jamileh Malakouti, Mahmoud Farvareshi, Mojgan Mirghafourvand

**Affiliations:** 1 Department of midwifery, Faculty of Nursing and Midwifery, Tabriz University of medical sciences, Tabriz, Iran; 2 Midwifery Department, Tabriz University of Medical Sciences, Tabriz, Iran; 3 Clinical Psychologist, Razi Hospital, Tabriz University of Medical Sciences, Tabriz, Iran; 4 Social Determinants of Health Research Center, Faculty of Nursing and Midwifery, Tabriz University of Medical Sciences, Shariati Street, P.O. Box: 51745-347, Tabriz, 513897977 Iran; 5 Department of Family Health, Social Determinants of Health Research Center, Ardabil University of Medical Sciences, Ardabil, Iran

**Keywords:** Anxiety, Stress, Depression, Quality of life, Sleep quality, Acceptance and commitment therapy, Counseling

## Abstract

**Background:**

One of the most critical periods in a woman’s life is menopause. During menopause, depression and anxiety are among the most common mood changes. Sleep disorders also increase during menopause, which leads to quality of life disorders. Different methods such as medication, psychotherapy, or a combination of them are used to treat these disorders. Acceptance and commitment-based therapy is one of the newest methods in psychotherapy that recently has been used a lot. Therefore, this study was conducted to determine the effect of acceptance and commitment therapy (ACT) on mood (primary outcome), sleep quality, and quality of life (secondary outcomes) of menopausal women.

**Methods:**

This randomized controlled trial was performed on 86 menopausal women in Tabriz, Iran in 2021. Using the blocking method, participants were randomly assigned into the intervention and control groups. The intervention group received counseling based on ACT approach in 8 sessions of 60 to 90 min. The control group received only routine health care. Depression, Anxiety, Stress Scale-21 (DASS 21), Menopause Quality of Life (MENQOL), and Pittsburgh Sleep Quality Index (PSQI) questionnaires were completed before intervention and immediately after the intervention. Independent t-test and Mann-Whitney U test were used to compare the outcomes between the two groups.

**Results:**

In terms of sociodemographic characteristics and baseline values of the studied variables, there was no statistically significant difference between the study groups before the intervention. At the end of the intervention, the mean (SD: standard deviation) scores of anxiety, stress, and depression in the counseling group were 2.66 (1.28), 2.91 (1.62), and 1.98 (1.59) and in the control group were 4.19 (1.85), 5.61 (1.49) and 3.59 (1.91). In the intervention group, the mean score of all three variables was significantly lower than the control group (*P* < 0.001). After the intervention, the mean (SD) of the total sleep quality score was 4.04 (2.52) in the counseling group and 4.13 (2.63) in the control group. In addition, the mean (SD) of the total quality of life score was 23.47 (20.13) in the counseling group and 23.14 (17.76) in the control group. Between the study groups, there were no statistically significant differences in the mean of the overall score of sleep quality (*P* = 0.867) and the overall score of quality of life (*P* = 0.759).

**Conclusion:**

Using ACT-based counseling can improve the mood of menopausal women. However, further randomized clinical trials are needed before making a definitive conclusions.

**Trial registration:**

Iranian Registry of Clinical Trials (IRCT): IRCT20120718010324N65. Date of registration: 2/19/2021. Date of first registration: 2/19/2021. URL: https://en.irct.ir/user/trial/53544/view; Date of recruitment start date: 2/22/2021.

## Background

Women experience different periods during their lives and developmental stages. One of the most critical periods in a woman’s life is menopause [1]. Menopause is a natural process that is shown by the permanent interruption of the menstrual cycle due to the loss of ovarian function [2]. It takes 2 to 8 years for women to go from premenopausal to menopausal. Due to changes in ovarian function, menstrual cycles in women change during this time. In addition, the hormonal-nervous system that controls ovulation in these women begins to secrete hormones irregularly and at long intervals. Although the main sign of menopause is high follicular stimulating hormone (FSH) levels, Gonadotropin levels remain high during this period [3]. Menopause happens approximately at the age of 50 [4]. The average age of menopause varies by race, genetics, nutrition, physical activity, and sexual behavior [5, 6]. The average age of menopause in urban, rural, and total population of Iran is respectively 49.9, 49.2, and 49.6 years [7].

A wide range of physiological, anatomical, and clinical changes appear in women due to the decreased ovarian function with declining levels of hormones, including estrogen and androgens that vary from woman to woman [8]. In different people, these symptoms can appear as minor discomfort to severe and debilitating symptoms [1]. These symptoms include flushing, menstrual irregularities, feeling of urgency in urination, bladder inflammation, predisposition to urinary tract infections, breast tenderness, vaginal dryness and atrophy, sleep disorders, sexual dysfunction, night sweats, osteoporosis, and other disorders such as depression, anxiety, anger, fatigue and irritability [9, 10].

Hormonal changes during menopause may affect mood during this period, for example, depressive and anxiety symptoms have been described among postmenopausal women [11]. According to studies, this mood is changed due to changes in estradiol levels and the association of serum estrogen levels with levels of platelet monoamine oxidase, which is a marker of adrenergic and serotonergic function [12]. In other words, due to decreased self-confidence, lack of activity and mobility, loss of friends and relatives, decreased physical and material independence, and chronic diseases, menopausal women are more exposed to anxiety [13]. Anxiety was independently associated with low quality of life and severe menopausal symptoms based on a study about postmenopausal women [14]. In addition to hormonal changes, other factors are effective in causing and exacerbating mood changes. These factors are the adverse effect of vasomotor symptoms on mood (domino theory), bad events in social life such as illness, previous history of depression, retirement, death of a spouse, care of elderly parents, empty nest syndrome, how women feel about menopause, prolonged menopause, chronic pain and disability, changes in sexual function, the level of emotional intelligence, and menopausal symptoms such as flushing, night sweats, and secondary sleep disorders can also affect mood changes [15, 16]. It is reported that the prevalence of sleep problems is up to 65% in menopausal women. Sleep disorders during menopause cause quality of life disorders [17]. It also decreases the ability to perform physical, mental, and social daily activities and affects mood changes in women [18].

Over 75% of women search for strategies and treatments to reduce menopausal symptoms [19]. Different methods are used to treat these disorders such as medication, psychotherapy, or a combination of them. To reduce flushing, sexual disorders and vaginal dryness, night sweats, restlessness, and urinary disorders, nearly, 40% of women use medical treatments [20]. Hormone therapy (HRT) is the most effective treatment for vasomotor symptoms of menopause among the pharmacologic therapies so that it treats 80 to 90% of the symptoms [21]. Due to an increased risk of breast cancer, pulmonary embolism, and cardiovascular disease, the Women’s Health Initiative has banned the use of the hormone in menopausal women [2]. Therefore, HRT is not recommended as a first-line treatment for menopausal women due to the complications and risks. Other therapies, including complementary therapies, herbal remedies, pharmacologic therapies, and behavioral modification can be as effective as HRT. It can treat the menopausal vasomotor symptoms [22]. Acceptance and commitment therapy (ACT) is one of the newest methods in psychotherapy which has come to the attention. Psychological interventions are used to help individuals to manage the social, emotional, and psychological challenges [23].

ACT dates back to a philosophical theory called functional contextualism. It is performed based on a research program on language and cognition called the theory of the framework of mental relations. ACT has six central processes that lead to psychological flexibility. These six processes include acceptance, diffusion, self as context, communication with the present, values, and committed action [24]. The theory of communication systems is the basis of this treatment. According to this theory, there are many ways to solve our problems but they trap us which leads to our resentment. Avoiding stress and anxiety are considered as the main problem of patients in this treatment, which leads to disability and reduced life satisfaction. According to this theory, when negative thoughts and emotions have an excessive and inappropriate effect on behavior, avoidance occurs. Therefore, exposing the patient to situations that are mostly avoided is the main method of treating ACT [25–27].

Research to date has supported the effectiveness of ACT for the improvement of depression [28, 29], anxiety [28, 30], stress [29, 30], general mental health [30], insomnia and sleep quality [31], and quality of life [27, 31] among different populations such as unemployed individuals, women with multiple sclerosis, mothers of children with hearing loss, patients with hypertension, social workers, women with breast cancer and pregnant women. Only a research have assessed the effect of ACT on sexual function in menopausal women [32] and no study focused on the effect of ACT on mood, sleep quality and quality of life in menopausal women. Even though, some studies showed the positive effect of other similar methods used in psychology such as cognitive therapy in improving depression, anxiety, insomnia and quality of life among menopausal woman [33, 34].

Women spend more than a third of their lives during menopause due to the increasing trend of elderly in the world and Iran and increasing life expectancy [35]. These biological and endocrine changes affect a woman’s sense of physical and mental health [36, 37]. Therefore, it is important to pay attention to the health of this period and improve the quality of life. On the other hand, depression and anxiety are among the common mood changes during menopause [12]. Due to the side effects of long-term use of drug treatments, alternative psychological therapies can be used [38]. In addition to effectiveness, counseling in this field will reduce overall health costs [39, 40]. Therefore, this study was conducted to determine the effectiveness of treatment based on acceptance and commitment on mood (primary outcome) and quality of life and sleep quality (secondary outcomes) in menopausal women.

### Study hypotheses

1. The mean depression score is significantly lower in the counseling group compared to the control group.
2. The mean anxiety score is significantly lower in the counseling group compared to the control group.
3. The mean stress score is significantly lower in the counseling group compared to the control group.
4. The mean quality of life score is significantly lower in the counseling group compared to the control group.
5. The mean sleep quality is significantly lower in the counseling group compared to the control group.

## Methods

### Study design and participants

This randomized controlled trial with two parallel groups was performed on 86 postmenopausal women in Tabriz-Iran health centers from March to the end of August 2021.

The inclusion criteria included willingness to participate in the study, having normal menopause, being married, having at least a middle school education, women with less than 10 years of menopause, earning an anxiety score of 4–8 (mild and moderate anxiety), depression score between 5 and 11 (mild and moderate depression), stress score between 8 and 13 (mild and moderate stress) according to DASS questionnaire, having a minimum of 45 years and a maximum of 60. There are also exclusion criteria such as the use of tobacco and alcoholic beverages and herbal medicines, known systemic problems including cardiovascular, gastrointestinal, liver, blood, endocrine, etc., use of any effective drug against flushing (clonidine, methyldopa, gabapentin, selective serotonin reuptake inhibitors, norepinephrine inhibitors, soy isoflavones), participating in relaxation and yoga classes and not using anti-anxiety drugs, not using sedatives including cinnamon and chamomile.

Based on the results of the study of Shariat Moghani et al. [41], the sample size was calculated 39 people by using the G-power software [42] for stress variable with considering the M_1_ = 7.07 (mean score of stress subscale of DASS), M_2_ = 4.59 (assuming 35% reduction due to intervention), SD_1_ = SD_2_ = 4.33, one-sided α = 0.05, and Power = 80%. There were 43 people in each group in the final sample size considering the 10% attrition.

### Sampling

The researcher referred to the health centers of Tabriz for sampling. Then, a list of all women around menopausal ages (45 to 60 years) with their phone numbers and addresses were extracted, menopausal women were called, the goals and methods of the study were briefly explained, and the women were examined for inclusion and exclusion criteria. Due to the spread of the coronavirus and compliance with health protocols, they were also asked to be online in the WhatsApp program at a certain time if they are eligible and willing to participate in the study. The objectives of the research were fully explained in the online session. Written informed consent was obtained in a face to face session if the individual tended to participate in the study, then the Depression, Anxiety, Stress Scale-21 (DASS 21) was completed through interviews with participants.

Menopausal women completed the questionnaire of socio-demographic and obstetric characteristics, Menopause Quality of Life (MENQOL) Questionnaire and Pittsburgh Sleep Quality Index (PSQI Index) through interviews if they obtained anxiety score 4 to 8 (mild and moderate anxiety), depression score 5 to 11 (mild and moderate depression), and stress score 8 to 13 (mild and moderate stress). Menopausal women with severe and very severe depression, stress, and anxiety were referred to a psychiatrist.

### Randomization

By using a random blocking method with a 1: 1 allocation ratio, participants were allocated to the intervention (counseling) and control groups. By using Randomiser software [43], the allocation sequence was determined by a person who did not involve in the study and was not aware of the study process. The type of intervention was written on paper and placed in opaque and sealed envelopes that numbered sequentially to conceal the allocation sequence. The envelopes were opened in the order in which the participants entered the study and the type of group of individuals was determined.

### Intervention

For the intervention group, the researcher (first author) held counseling sessions. The counseling sessions were held face to face in a place intended for counseling with a quiet and private environment in health centers. There were 8 counseling sessions. During the sessions, women were given the necessary information, and there were interactive sessions between the counselor and the women. Counseling was performed with an approach based on ACT, during 60–90 min sessions and one session per week, in groups of 8–12 people. It was also held in a place with a suitable space to observe the social distance, proper air conditioning and the observance of health protocols. No one was infected with Covid-19 during study.

Here is the treatment plan of the counseling group meetings:

Session 1: Greeting with members, description of rules and duties of group members, statement of goals, the introduction of counseling based on ACT. General assessment: the main complaint of the participants, identifying the experimental avoidances of the participants, identifying thoughts and feelings behind these actions, identifying the strengths of the participants.

Session 2: Brief description of the anatomy and physiology of the reproductive system (uterus, ovaries, etc.), the definition of menopause, explanation of menopausal symptoms and their effects on various aspects of life, available drug treatments, and non-drugs methods and their success rates.

Session 3: Teaching mindfulness techniques.

Session 4: The technique of frustration using the metaphor of wells and shovels, the practice of suppressing the mind to show the uncontrollability of the mind; Homework: self-monitoring.

Session 5: Assessing homework, teaching the concept of acceptance: using the metaphor of the guest and teaching the concept of the self-observer. Techniques for not taking seriously the thoughts and practicing them: watching thoughts, taking on the role of a TV reporter, or singing thoughts.

Session 6: Introducing the concept of values and committed actions and the difference between values and purpose and introducing the list of values, using the metaphor of birthday party. Homework: Completing the worksheet of goals, values, and effective actions and obstacles.

Session 7: Start the session with contact with the present moment exercises, control, and evaluation of worksheets, introduction, and training of cognitive inconsistencies, using the metaphor of bus.

Session 8: Repetition of mindfulness techniques and retrieval of assignments and review of exercises.

No intervention was applied to the control group and they received only routine care included: control of blood pressure and weight gain, control hot flushes and other menopausal symptoms, breast exam, pelvic exam, prescription to undergoing mammography and Papsmear test [44]. Also, after the completion of the project, the content of the counseling sessions was provided to the participants of the control group.

### Data collection tools

To collect data, the socio-demographic and obstetrics characteristics questionnaire, DASS 21, MENQOL and PSQI were used in this study. The researcher completed the questionnaire before and immediately after the intervention through interviews with participants.

### Socio-demographic and obstetrics characteristics questionnaire

This questionnaire includes questions such as age, duration of menopause, menopausal age, level of education, occupation, level of education and occupation of spouse and adequacy of family monthly income for living expenses, number of family members, the relation of people living with participant (living with the daughter-in-law and son-in-law), body mass index (BMI), cigarette smoking in the participant and her spouse and life satisfaction. Content and face validity were used to determine the validity of the socio-demographic and obstetrics characteristics questionnaire. The questionnaire was given to the faculty members. Based on the feedback received, the corrections were made on the tools after collecting their opinions.

### MENQOL

In this study, to measure specific criteria for the quality of life of postmenopausal women, the MENQOL questionnaire was used. The medical department of the University of Toronto, Canada (1996) prepared and used this questionnaire. MENQOL has been previously used in Iran and in the Yazdkhasti et al.’s study [45], the test-retest analysis method was used to determine the reliability this questionnaire and correlation coefficient (r) was reported equal to 0.84. The questionnaire has 29 closed questions with a rating range of zero to six based on the Likert scale. It includes 4 dimensions, vasomotor (3 questions), psychosocial (7 questions), physical (16 questions), and sexual (3 questions). The quality of life score of menopausal women is calculated from the scores obtained from these 4 subdomains. The minimum and maximum scores obtained in the vasomotor dimension are 0 to 18, psychosocial 0 to 42, physical 0 to 96, and sexual 0 to 18 based on how to score on a 6-point Likert scale and the number of questions available. According to mentioned dimensions, the total score of quality of life is from 0 to 174. High scores indicate more severe menopausal symptoms and lower quality of life in postmenopausal women [45].

### PSQI

This questionnaire includes 18 questions and seven components including subjective sleep quality, sleep latency, sleep duration, sleep efficacy, sleep disturbances, use of sleep medication and daytime dysfunction. The score of each question is between 0 and 3 and the score of each component is a maximum of 3. The sum of the average scores of these seven components is the total score of the instrument, which ranges from 0 to 21. The quality of sleep is lower if the score obtained becomes high. A score higher than 5 indicates poor sleep quality [46]. The Iranian version of this questionnaire is a valid and reliable tool for measuring sleep quality. According to Nazifi et al., the reliability of this instrument with Cronbach’s alpha was reported to be 0.55 [47].

### DASS

This scale includes three subscales of stress, depression, and anxiety. This questionnaire consists of 21 questions. For each of the three subscales, 7 questions are considered [48, 49]. The scoring for each question is based on the Likert scale from never (0) to very high [3]. The score is calculated for each scale separately and the overall score is not calculated. For each subscale, the minimum score is zero and the maximum is 21 and a higher score indicates a worse situation [50]. The DASS 21 scale can diagnose and screen symptoms of anxiety, depression, and stress over the past week. Menopausal women aged 45–60 years entered the study who received an anxiety score of 4–8 (mild and moderate anxiety), a depression score between 5 to 11 (mild and moderate depression), and a stress score between 8 to13 (mild and moderate stress) from DASS questionnaire. This questionnaire was also validated In Iran and its validity was reported for anxiety subscale 0.73, depression subscale 0.81, and stress subscale 0.81 [51].

### Data analysis

The data were analyzed using SPSS-Version 24 software after collecting information from all participants. The normality of quantitative data was assessed using the Kolmogorov – Smirnov test. Chi-square, chi-square for trend, independent t, and Fisher’s exact tests were used to compare socio-demographic and obstetrics characteristics between the two groups. An independent t-test was used to compare the mean score of variables with normal distribution among study groups. Mann-Whitney U test was used for variables with the non-normal distribution. Within group comparisons were conducted using the Wilcoxon test for variables with the non-normal distribution and paired samples t-test for variables with normal distribution.

All tests were performed based on Intention-To-Treat. *P* < 0.05 was considered significant.

## Results

**Fig. 1:**
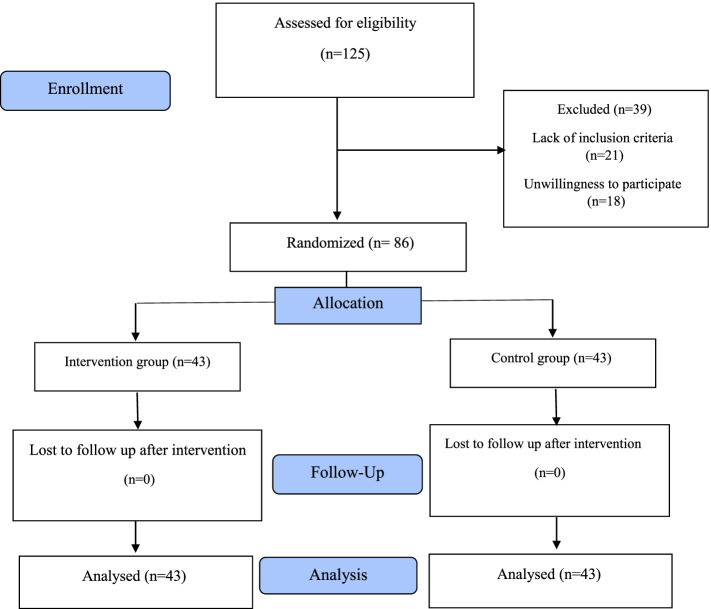
Flow chart of the study

Sampling started in March 2020 and continued until August 2021. The researcher evaluated 125 women aged 45 to 60 years and 86 of them qualified the inclusion criteria. 43 women were allocated randomly to the counseling group, and they participated in 8 counseling sessions. There was no drop in the study. 86 women were retested and the data were analyzed after the intervention (Fig. 1).

In the intervention group, the mean (SD: standard deviation) age of the counseling group was 54.87 (4.49) and in the control group was 54.75 (4.65) years old. Table 1 shows the socio-demographic and obstetrics characteristics of participants.

**Table 1:** Sociodemographic and obstetrics characteristics in study groups

variable	Counseling group (***n*** = 43)	Control group (***n*** = 43)	***P***-value
	Mean (SD^⁕^)	Mean (SD^⁕^)	
**Age** (Year)	54.87 (4.49)	54.75 (4.56)	0.905^†^
**Menopausal age** (Year)	49.77 (3.03)	50.40 (3.09)	0.345^†^
**Body mass index** (kg/m2)	29.45 (5.38)	29.13 (5.44)	0.786^†^
	Number (percent)	Number (percent)	
**Education**			0.375^§^
Secondary school	28 (65.1)	25 (58.1)	
Highschool	2 (4.7)	3 (7.0)	
Diploma	11 (25.6)	9 (20.9)	
University	2 (4.7)	6 (14.0)	
**Job**			0.534^‡^
Housewife	38 (88.4)	36 (83.7)	
Employed	5 (11.6)	7 (16.3)	
**Housband education**			0.746^§^
Iilliterate	3 (7.0)	5 (11.6)	
Elementary school	5 (11.6)	5 (11.6)	
Secondary school	12 (27.9)	11 (25.6)	
Highschool	5 (11.6)	1 (2.3)	
Diploma	11 (25.6)	7 (16.3)	
University	7 (16.3)	14 (32.6)	
**Housband job**			0.199^‡^
Free job	16 (37.2)	7 (16.3)	
Employed	9 (20.9)	12 (27.9)	
Retired	14 (32.6)	19 (44.2)	
Unemployed	4 (9.3)	4 (9.3)	
Laborer	0 (0.0)	1 (2.3)	
**Parity**			0.273^‡^
One	13 (30.2)	20 (46.5)	
Two	20 (46.5)	14 (32.6)	
Three	10 (23.3)	9 (20.9)	
**Number of children**			0.376^‡^
One	18 (41.9)	23 (53.3)	
Two	17 (39.5)	11 (25.6)	
Three	8 (18.6)	9 (20.9)	
**Sufficiency of family income for expenses**			0.417^§^
Completely sufficient	1 (2.3)	2 (4.7)	
Relatively sufficient	40 (93.0)	40 (93.0)	
Insufficient	2 (4.7)	1 (2.3)	
**Number of family members**			0.053^‡^
1 to 3	38 (88.4)	30 (69.8)	
4 to 6	5 (11.6)	10 (23.3)	
Over 6	0 (0.0)	3 (7.0)	
**Living with which member**			0.156^‡^
Daughter-in-law	2 (4.7)	6 (14.0)	
Son-in-law	0 (0.0)	1 (2.3)	
None	41 (95.3)	36 (83.7)	
**Satisfaction of life**			1.000^§^
Satisfied	41 (95.3)	40 (93.0)	
Not Satisfied	0 (0.0)	2 (4.7)	
No idea	2 (4.7)	1 (2.3)	
**Husband smoking**			0.610^‡^
Yes	9 (20.9)	11 (25.6)	
No	34 (79.1)	32 (74.4)	

*Standard Deviation; ^†^Independent t-test; ^‡^Fisher’s exact test; ^§^Chi-square test for trend

Before intervention, based on the Mann-Whitney U test, there was no statistically significant difference between the two groups in terms of anxiety (*P* = 0.189), stress (*P* = 0.878) and depression (*P* = 0.611). However, after the intervention, the mean score of anxiety, stress and depression was significantly lower in the counseling group than the control group (*P* < 0.001) (Table 2).

**Table 2:** Comparison of stress, anxiety and depression between study groups

Variable	Counseling group (***n*** = 43)		Control group (***n*** = 43)		***P***-value
	Mean (SD^‡^)	Median (Per 25 to 75)^§^	Mean (SD^‡^)	Median (Per 25 to 75)^¥^	
**Depression**					
Before intervention	6.24 (1.88)	6.00 (5.00 to 7.00)	6.07 (1.57)	6.00 (5.00 to 7.00)	0.611^*^
After intervention	1.98 (1.59)	2.00 (1.00 to 3.00)	3.59 (1.91)	3.00 (2.00 to 5.00)	< 0.001^*^
Intra-group comparison (*P*-value)^¥^	< 0.001		< 0.001		
**Anxiety**					
Before intervention	6.89 (1.82)	7.00 (6.00 to 8.00)	6.38 (1.47)	6.00 (5.00 to 8.00)	0.189^*^
After intervention	2.66 (1.28)	3.00 (2.00 to 4.00)	4.19 (1.85)	4.00 (2.00 to 6.00)	< 0.001^†^
Intra-group comparison (*P*-value)^¥^	< 0.001		< 0.001		
**Stress**					
Before intervention	8.73 (1.55)	9.00 (8.00 to 9.00)	8.89 (1.08)	9.00 (8.00 to 9.00)	0.878^*^
After intervention	2.91 (1.62)	3.00 (2.00 to 4.00)	5.61 (1.49)	5.00 (5.00 to 7.00)	< 0.001^†^
Intra-group comparison (*P*-value)^¥^	< 0.001		< 0.001		

^*^Mann Whitney U test; ^†^Independent t-test; ^‡^Mean (Standard Deviation); ^§^Median (Percentile 25 to Percentile 75);

^¥^ Wilcoxon test

According to the Mann-Whitney U test, there was no statistically significant difference between the two groups before (*P* = 0.548) and after the intervention (*P* = 0.867). There was no statistically significant difference between the groups in terms of sleep quality subdomains (*P* > 0.05) (Table 3).

**Table 3:** Comparison of sleep quality and its subscales between study groups

Variable	Counseling group (***n*** = 43)		Control group (***n*** = 43)		***P***-value
	Mean (SD^‡^)	Median (Per 25 to 75)^§^	Mean (SD^‡^)	Median (Per 25 to 75)^§^	
**Subjective sleep quality**					
Before intervention	0.96 (0.85)	1.00 (0.00 to 1.00)	0.68 (0.72)	1.00 (0.00 to 1.00)	0.130^*^
After intervention	0.54 (0.67)	0.00 (0.00 to 1.00)	0.56 (0.71)	0.00 (0.00 to 1.00)	0.926^*^
Intra-group comparison (*P*-value)^¥^	< 0.001		0.025		
**Sleep latency**					
Before intervention	1.37 (1.04)	1.50 (0.50 to 2.50)	1.41 (1.05)	1.50 (0.00 to 2.50)	0.837^†^
After intervention	0.88 (0.91)	0.50 (0.00 to 1.50)	1.09 (0.94)	1.00 (0.00 to 2.00)	0.334^*^
Intra-group comparison (*P*-value)^¥^	< 0.001		< 0.001		
**Sleep duration**					
Before intervention	0.98 (0.86)	1.00 (0.00 to 1.00)	0.94 (0.71)	1.00 (0.00 to 1.00)	0.985^*^
After intervention	0.70 (0.71)	1.00 (0.00 to 1.00)	0.70 (0.64)	1.00 (0.00 to 1.00)	0.920^*^
Intra-group comparison (*P*-value)^¥^	0.001		0.012		
**Sleep efficacy**					
Before intervention	0.14 (0.42)	0.00 (0.00 to 0.00)	0.17 (0.38)	0.00 (0.00 to 0.00)	0.570^*^
After intervention	0.05 (0.22)	0.00 (0.00 to 0.00)	0.05 (0.22)	0.00 (0.00 to 0.00)	1.000^*^
Intra-group comparison (*P*-value)^¥^	0.046		0.025		
**Sleep disturbance**					
Before intervention	1.26 (0.50)	1.00 (1.00 to 2.00)	1.17 (0.44)	1.00 (1.00 to 1.00)	0.339^*^
After intervention	1.17 (0.49)	1.00 (1.00 to 1.00)	1.05 (0.31)	1.00 (1.00 to 1.00)	0.165^*^
Intra-group comparison (*P*-value)^¥^	0.102		0.025		
**Use of sleeping medication**					
Before intervention	0.63 (1.03)	0.00 (0.00 to 2.00)	0.38 (0.66)	0.00 (0.00 to 1.00)	0.466^*^
After intervention	0.49 (0.91)	0.00 (0.00 to 1.00)	0.38 (0.66)	0.00 (0.00 to 1.00)	0.844^*^
Intra-group comparison (*P*-value)^¥^	0.034		1.000		
**Daytime dysfunction**					
Before intervention	0.31 (0.52)	0.00 (0.00 to 1.00)	0.52 (0.83)	0.00 (0.00 to 1.00)	0.350 ^*^
After intervention	0.24 (0.43)	0.00 (0.00 to 0.00)	0.33 (0.57)	0.00 (0.00 to 1.00)	0.546 ^*^
Intra-group comparison (*P*-value)^¥^	0.008		0.001		
**Total sleep quality score**					
Before intervention	5.62 (2.82)	5.50 (3.00 to 7.50)	5.23 (3.26)	4.00 (3.00 to 7.50)	0.548^†^
After intervention	4.04 (2.52)	4.00 (2.00 to 5.50)	4.13 (2.63)	3.50 (2.00 to 6.00)	0.867^†^
Intra-group comparison (*P*-value)^€^	< 0.001		< 0.001		

^*^Mann Whitney U test; ^†^Independent t-test; ^‡^Mean (Standard Deviation); ^§^Median (Percentile 25 to Percentile 75);

^¥^ Wilcoxon test; ^€^ Paired samples t-test

There was no statistically significant difference between the two groups before (*P* = 0.475) and after (*P* = 0.759) the intervention according to the Mann-Whitney U test. There was no statistically significant difference between the groups in terms of quality of life subdomains (*P* > 0.05) (Table 4).

**Table 4:** Comparison of quality of life and its subscales between study groups

Variable	Counseling group (***n*** = 43)		Control group (***n*** = 43)		***P***-value
	Mean (SD^‡^)	Median (Per 25 to 75)^§^	Mean (SD^‡^)	Median (Per 25 to 75)^§^	
**Vasomotor**					
Before intervention	6.07 (4.33)	6.00 (3.00 to 9.00)	5.26 (3.37)	4.00 (3.00 to 8.00)	0.333 ^†^
After intervention	4.07 (3.31)	3.00 (2.00 to 6.00)	4.45 (3.18)	4.00 (2.00 to 6.00)	0.451 ^*^
Intra-group comparison (*P*-value)^¥^	< 0.001		< 0.001		
**Psychosocial**					
Before intervention	5.87 (7.75)	4.00 (0.00 to 9.00)	3.77 (5.51)	1.00 (0.00 to 5.00)	0.222 ^*^
After intervention	3.66 (5.15)	2.00 (0.00 to 5.00)	3.12 (4.52)	1.00 (0.00 to 5.00)	0.435 ^*^
Intra-group comparison (*P*-value)^¥^	< 0.001		0.002		
**Physical**					
Before intervention	17.35 (13.57)	12.00 (9.00 to 24.00)	16.80 (13.35)	12.00 (8.00 to 16.00)	0.976^*^
After intervention	14.03 (12.3)	10.00 (5.00 to 18.00)	14.33 (11.88)	10.00 (7.00 to 14.00)	0.565^*^
Intra-group comparison (*P*-value)^¥^	< 0.001		< 0.001		
**Sexual**					
Before intervention	2.19 (4.14)	0.00 (0.00 to 2.00)	1.42 (2.89)	0.00 (0.00 to 1.00)	0.431 ^*^
After intervention	1.73 (3.44)	0.00 (0.00 to 1.00)	1.26 (2.66)	0.00 (0.00 to 1.00)	0.705 ^*^
Intra-group comparison (*P*-value)^¥^	0.004		0.176		
**Total quality of life score**					
Before intervention	31.47 (24.55)	24.00 (14.00 to42.00)	27.24 (19.99)	21.00 (13.00 to 29.00)	0.457^*^
After intervention	23.47 (20.13)	18.00 (9.00 to 28.00)	23.14 (17.76)	17.00 (10.00 to 26.00)	0.759^*^
Intra-group comparison (*P*-value)^¥^	< 0.001		< 0.001		

^*^Mann Whitney U test; ^†^Independent t-test; ^‡^Mean (Standard Deviation); ^§^Median (Percentile 25 to Percentile 75);

^¥^ Wilcoxon test

## Discussion

In our knowledge, this is the first study to examine the effect of ACT-based counseling on mood, sleep quality and quality of life among the menopausal women. According to the study results, in menopausal women, counseling based on ACT approach reduced anxiety, stress, and depression and improved their mood, but it did not affect sleep quality and quality of life.

In the present study, at the end of the intervention, the mean scores of stress, anxiety, and depression in the group receiving counseling based on ACT were significantly lower than the control group. Rajabi et al. (2014) assessed the effectiveness of ACT on anxiety and depression in women with multiple sclerosis. The results showed that ACT reduced anxiety, depression, and experiential avoidance of these women [28]. In addition, ACT reduced the stress and anxiety of Razi Psychiatric Center staff according to a study conducted by Heidari et al. (2018) [52]. Qarashi et al. (2019) conducted a study to investigate the effect of ACT on stress and anxiety of mothers of deaf or hard of hearing children. They showed that this intervention reduces mothers’ stress and anxiety [29]. Miri et al. (2021) examined the impact of the ACT counseling on stress and anxiety in patients with hypertension. In this study, the results showed that this intervention reduces stress and anxiety in hypertensive patients [30]. The results of all mentioned studies are consistent with the present study.

The ACT based counseling encourages clients to accept internal experiences. One of the main strategies of this intervention is acceptance. It is a choice that means leaving the discomfort with its reasons. Acceptance is an alternative process of control. It allows clients to accept unpleasant inner experiences without trying to control them [53]. In this case, experiences seem less threatening and have less effect on a person’s life. To help people gain control of their inner experiences, therapists use an acceptance and commitment-based approach with many metaphors and strategies [54].

The counseling did not affect the sleep quality of menopausal women in the present study. Rasouli et al. (2018) conducted a quasi-experimental study to investigate the effectiveness of acceptance and commitment-based psychotherapy in improving depression and sleep quality in women with postpartum depression. The results showed that commitment and acceptance-based therapy is significantly effective in reducing postpartum depression and increasing sleep quality [55]. Zakiei et al. (2021) conducted a study to investigate the effect of acceptance and commitment-based counseling on sleep quality, experimental avoidance, and emotion regulation in people with insomnia. It showed that this intervention improves sleep quality, experimental avoidance, and emotion regulation [56]. Salari et al. (2020) also conducted a study to determine the impact of acceptance and commitment-based intervention on sleep quality and insomnia. This study results also showed that ACT counseling improves sleep quality and insomnia [57].

In the present study, counseling did not affect the quality of life of postmenopausal women. Rezaei et al. (2020) conducted a quasi-experimental study to compare the efficacy of ACT-based counseling and compassion-focused treatment on psychological well-being and quality of life in patients with immunodeficiency virus. Findings indicated that both acceptance and commitment-based therapy and compassion-focused therapy had a positive effect on psychological well-being and quality of life in patients with immunodeficiency virus [58]. Samani et al. (2019) conducted also a study to compare the effect of ACT based counseling and physiotherapy on quality of life and pain catastrophizing. It was concluded that the quality of life in people receiving ACT-based counseling was significantly higher than those receiving physiotherapy. There was no difference in reducing the rate of pain catastrophizing in the two groups [59]. Hassani et al. (2018) conducted a study to determine the effect of the ACT based counseling on the quality of life and resilience of women with breast cancer. In this study, the results showed that the ACT-based intervention improved the quality of life and resilience of women with breast cancer [27]. Vakilian et al. (2019) conducted a study to determine the effect of ACT on quality of life and stress of pregnant women. The results showed that this intervention improved the quality of life and stress of pregnant women immediately after the intervention. But, this effect decreased 1 month after the intervention [31].

In the present study, the results are not consistent with the results of the above studies on the effect of ACT-based counseling on quality of sleep and life. This inconsistency may be attributed to variations of participants, and study time frame. The above mentioned studies have conducted on patients with immunodeficiency virus, women with breast cancer and pregnant women. The present study is the first study that assessed the effect of ACT-based counseling on quality of sleep and life among menopausal women. Numerous other factors affect the sleep quality of menopausal women such as neurological diseases, metabolic changes, weight gain and low levels of exercise, medications, lifestyle, and changes in hormone levels [60–62]. Also, in the present study, due to the concurrence of this study with the corona pandemic and its limitations such as quarantine and social distancing and the impact of this issue on different aspects of life, this intervention did not affect the quality of life and sleep. Quarantine in the COVID-19 epidemic has caused many changes in the lifestyle of most people [63]. In addition to the social and economic context, quarantine changed the cognitive and behavioral aspects of people’s lives [64]. These forced changes in lifestyle habits due to quarantine, such as changes in interpersonal relationships, eating habits, exercise, or sex are more common in postmenopausal women. They can exacerbate their menopausal symptoms and cause a loss of quality of life [65]. COVID-19 pandemic and quarantine have caused changes in the program, quality, and quantity of sleep such as going to bed late at night, waking up late in the morning, and reducing the quality of sleep and daily naps according to recent studies [66]. Some factors lead to circadian rhythm disturbances such as reduced exposure to sunlight, restriction of daily activities, and changes in meal times which can affect people’s sleep [67, 68].

### Strengths and limitations

One of the strengths of this study is observing all principles of randomized controlled trial, including random allocation and allocation concealment. Standard and valid questionnaires were used in this study, which the psychometric properties of these questionnaires have been assessed in Iran already. There was no attrition in this study. The limitation of this study was that it coincided with the coronavirus pandemic and severe restrictions due to social distancing and quarantine for counseling sessions. In addition, it was not possible to blind the participants and data assessor due to the nature of the intervention. Also, due to the non-normal distribution of data, the two-way ANOVA test wasn’t conducted. Other limitations are that sample size of this study was relatively small and the follow-up period was short. Therefore, it is recommended to conduct randomized clinical trials with larger sample size and longer follow-up period. Also, it is recommended that the similar study to be conducted after the end of coronavirus pandemic.

## Conclusion

One of the important goals of health care is promoting women’s mental health during menopause. Using ACT-based counseling can improve the mood of menopausal women. However, further randomized clinical trials are needed before making a definitive conclusion. Since, the counseling with ACT approach is easy, effective and understandable for women and also the healthcare services are provided free-of-charge in health centers of Iran, therefore, with the training of health care providers, they can improve the health of menopausal women by properly using of this approach.

## Data Availability

The datasets used and/or analysed during the current study available from the corresponding author on reasonable request.

## Abbreviations

ACT: Acceptance and Commitment Therapy
DASS 21: Depression, Anxiety Stress Scale-21
MENQOL: Menopause Quality of Life
PSQI: Pittsburgh Sleep Quality Index
SD: standard deviation
IRCT: Iranian Registry of Clinical Trials
FSH: Follicular Stimulating Hormone
HRT: Hormone Therapy
BMI: Body Mass Index
